# A pragmatic context assessment tool (pCAT): using a Think Aloud method to develop an assessment of contextual barriers to change

**DOI:** 10.1186/s43058-022-00380-5

**Published:** 2023-01-11

**Authors:** Claire H. Robinson, Laura J. Damschroder

**Affiliations:** VA Center for Clinical Management Research, VA Ann Arbor Healthcare System, 2215 Fuller Road (152), Ann Arbor, MI 48105 USA

**Keywords:** Qualitative, Implementation, Quality improvement, Think Aloud, Implementation science

## Abstract

**Background:**

The Consolidated Framework for Implementation Research (CFIR) is a determinant framework that can be used to guide context assessment prior to implementing change. Though a few quantitative measurement instruments have been developed based on the CFIR, most assessments using the CFIR have relied on qualitative methods. One challenge to measurement is to translate conceptual constructs which are often described using highly abstract, technical language into lay language that is clear, concise, and meaningful. The purpose of this paper is to document methods to develop a freely available pragmatic context assessment tool (pCAT). The pCAT is based on the CFIR and designed for frontline quality improvement teams as an abbreviated assessment of local facilitators and barriers in a clinical setting.

**Methods:**

Twenty-seven interviews using the Think Aloud method (asking participants to verbalize thoughts as they respond to assessment questions) were conducted with frontline employees to improve a pilot version of the pCAT. Interviews were recorded and transcribed verbatim; the CFIR guided coding and analyses.

**Results:**

Participants identified several areas where language in the pCAT needed to be modified, clarified, or allow more nuance to increase usefulness for frontline employees. Participants found it easier to respond to questions when they had a recent, specific project in mind. Potential barriers and facilitators tend to be unique to each specific improvement. Participants also identified missing concepts or that were conflated, leading to refinements that made the pCAT more understandable, accurate, and useful.

**Conclusions:**

The pCAT is designed to be practical, using everyday language familiar to frontline employees. The pCAT is short (14 items), freely available, does not require research expertise or experience. It is designed to draw on the knowledge of individuals most familiar with their own clinical context. The pCAT has been available online for approximately two years and has generated a relatively high level of interest indicating potential usefulness of the tool.

**Supplementary Information:**

The online version contains supplementary material available at 10.1186/s43058-022-00380-5.

Contributions to the literature

- Context assessment is a core function within implementation projects, but it is challenging to translate concepts that often use highly abstract, technical language into everyday language that is clear, concise, and meaningful to frontline clinicians.
- We developed an abbreviated pragmatic context assessment tool (pCAT) that is short (14 items), freely available, uses accessible language, does not require research expertise or experience.
- The pCAT is a practical tool that can be used by researchers or frontline teams as an abbreviated assessment of common barriers and facilitators in local clinical contexts.

## Background

Implementation scientists recognize that determinants (barriers or facilitators) within local context impact implementation efforts. Assessing context before, during, and/or after implementation is important so that implementers can use this information identify optimal strategies that can be used to address barriers and leverage facilitators [1]. Easy-to-use quantitative context assessment tools rooted in the concepts and evidence-base within implementation science need to be developed. Such tools rely on frontline clinicians and staff accurately understanding of what is being asked within assessment instruments. However, these individuals are often not familiar with the language used in these assessments or how it applies to their own situation. Assessments should be rooted in theoretical constructs and yet also need to be conceptually clear using every-day language.

The Consolidated Framework for Implementation Research (CFIR) is a determinant framework, designed to identify barriers and facilitators that potentially impact implementation outcomes. Though frameworks like the CFIR seek to provide clarity and consistency in terms and definitions for each construct, the language used can be highly technical. The dominant approach for identifying barriers and facilitators has relied on researchers conducting assessments based on information elicited through qualitative interviews that are analyzed, interpreted, and used to develop tailored strategies with guidance for local practitioners to help them navigate their context for successful implementation [1–5]. Measurement instruments seek to elicit quantitative assessments of barriers and facilitators because this can be a more efficient way to assess context. However, these instruments are often exceedingly long or require expertise and training to use [6–11]. Frontline clinicians and staff who do the work of implementation may misunderstand or misapply questions designed to elicit potential barriers and facilitators; they are often more familiar with quality improvement language [12–16].

Pragmatic measures of context are needed. Glasgow and Riley define pragmatic measures as being important to stakeholders, low burden (usually indicated by a low number of survey items), actionable, and sensitive to change [17]. Stanick et al. add that pragmatic measures are feasible, low cost, and brief [18]. Guided by these principles, an abbreviated pragmatic context assessment tool (pCAT) was developed based on the CFIR. This instrument has been available online (www.CFIRguide.org) and has generated a high level of interest, generating nearly 50 requests over approximately 18 months (2021–2022). Thus, the purpose of this paper is to document methods used to develop the pCAT.

## Methods

Our research team developed an abbreviated context assessment tool based on CFIR constructs that repeatedly arose as potential barriers or facilitators in implementation [19–23]. This tool was piloted with six frontline improvement teams (see Table 1); the teams collectively comprised 21 individuals who participated in the Learn. Engage. Act. Process. (LEAP) Program [23]. LEAP is a 26-week, virtual, coach-led, structured learning program designed to develop competency in the application of quality improvement methods and techniques for frontline clinicians and staff. The goal was for teams to use the assessment tool to identify potential barriers and facilitators to implementing improvements, so they could better understand the micro-level context within which they were working to improve processes and programs. We had concerns with the piloted version, however, because many responses did not reflect actual barriers and facilitators observed by and reported to the LEAP coaches who worked closely with frontline teams. We took the opportunity to pause, reflect, and update the pCAT.

**Table 1:** List of CFIR constructs included in Think Aloud survey development

Survey introduction			Pilot version		Version used in initial Think Aloud interviews^a^	Final version of pCAT
**Question Stem** (open-text response)**:**			Please enter your problem area (area for improvement). This should reflect whatever topic you and your team are currently considering. It does not have to be final (e.g., The majority of patients fail to show up for scheduled orientation)		Please enter your problem area (area for improvement). This should reflect whatever topic you and your team are currently considering. It does not have to be final (e.g., The majority of patients fail to show up for scheduled orientation)	We’ve found that it’s best to think concretely about a planned or on-going implementation (as opposed to the more general implementation environment). Include the specifics of the implementation/improvement project here
**Identifying Barriers vs. Facilitators** (item responses):			Overall, we are most likely to encounter more (check only one): 1 = Facilitating forces related to [construct] 2 = Hindering forces related to [construct] 3 = We cannot think of any facilitating or hindering forces related to [construct]		1 = Disagree 2 = Neutral 3 = Agree	1 = Disagree: This means the item is a potential barrier 2 = Neutral 3 = Agree: This means the item is a potential facilitator
**Effect on implementation** (item responses):			1 = Weak 2 = Moderate 3 = High		1 = Low impact 2 = Moderate impact 3 = High impact	0 = Weak/no effect 1 = Strong effect
**CFIR-based survey items**						
**CFIR domain**	**CFIR construct**	**Short definition**		**Survey version 1.0**	**Survey version 2.0**	**Final survey**
Program Characteristics	Relative Advantage	Stakeholders’ perception of the advantage of implementing the intervention versus an alternative solution		*The extent to which leaders and staff recognize the potential benefit of implementing a change to address your problem area, especially compared to other alternatives, may influence your success*	*Key people will see the advantage of implementing the change versus an alternative*	*People here see the advantage of implementing the change versus an alternative*
Outer Setting	Patient Needs & Resources	The extent to which patient needs, as well as barriers and facilitators to meet those needs are accurately known and prioritized by the organization		*The extent people are aware of Veteran needs and preferences and use that information to improve programs may influence your success*	*People here regularly seek to understand the needs of patients and make changes to better meet those needs*	**[NO CHANGE from version used in Think Aloud interviews]** *People here regularly seek to understand the needs of patients and make changes to better meet those needs*
Inner Setting	Structural Characteristics	The social architecture, age, maturity, and size of an organization		*The structural characteristics, including the age, size, how people and processes are organized, hierarchical structure, policies and procedures may influence your success*	*The structures and policies in place here will enable us to make the change*	**[NO CHANGE from version used in Think Aloud interviews]** *The structures and policies in place here enable us to make the change*
	Networks & Communications	The nature and quality of webs of social networks and the nature and quality of formal and informal communications within an organization		*Quality and type of working relationships between people and the quality of communications may influence your success*	*I have open lines of communication with everyone needed to make the change*	**[NO CHANGE from version used in Think Aloud interviews]** *I have open lines of communication with everyone needed to make the change*
	Tension for Change	The degree to which stakeholders perceive the current situation as intolerable or needing change		*The degree to which stakeholders perceive the current situation as intolerable or needing change may influence your success*	*Key people will see the current situation as intolerable and that the change is needed*	*People here see the current situation as intolerable and that the change is needed*
	Compatibility	The degree of tangible fit between meaning and values attached to the intervention by involved individuals, how those align with individuals’ own norms, values, and perceived risks and needs, and how the intervention fits with existing workflows and systems		*The level of compatibility between the change you might implement and (1) existing workflows and systems; and (2) norms, values, and perceived risks and needs in your facility, may influence your success*	*The change is compatible with existing clinical processes*	**[NO CHANGE from version used in Think Aloud interviews]** *The change is compatible with existing clinical processes*
					*The change is aligned with clinician values related to weight loss among Veterans*	**[NO CHANGE from version used in Think Aloud interviews]** *The change is aligned with clinician values related to weight loss among Veterans*
	Goals & Feedback	The degree to which goals are clearly communicated, acted upon, and fed back to staff and alignment of that feedback with goals		*The extent leaders clearly communicate goals and provide helpful feedback, as well as the extent feedback aligns with organizational and clinical goals, may influence your success*	*The change is aligned with leadership goals*	***[NO CHANGE from* version used in Think Aloud interviews*]*** *The change is aligned with leadership goals*
	Learning Climate	A climate in which: (a) leaders express their own fallibility and need for team members’ assistance and input; (b) team members feel that they are essential, valued, and knowledgeable partners in the change process; (c) individuals feel psychologically safe to try new methods; and (d) there is sufficient time and space for reflective thinking and evaluation		*The extent to which people feel encouraged to experiment, look for ways to improve current processes and programs, and learn from mistakes may influence your success*	*Not assessed*	*Not assessed*
	Leadership Engagement	Commitment, involvement, and accountability of leaders and managers with the implementation		*The level of commitment and involvement of leaders in your charter aim may influence your success*	*Leaders here will be committed, involved, and accountable for the planned improvement*	*Higher level leaders are committed, involved, and accountable for the planned improvement*
						*Leaders I work with most closely are committed, involved, and accountable for the planned improvement*
	Available Resources	The level of resources dedicated for implementation and on-going operations including money, training, education, physical space, and time		*The level of resources available to support your charter aim, including money, physical space, and time, may influence your success*	*We will have sufficient time dedicated to make the change*	*We have sufficient time dedicated to make the change*
					*We will have sufficient space to accommodate the change*	*We have sufficient space to accommodate the change*
					*We will have other needed resources to make the change*	*We have other needed resources to make the change*
Process	Reflecting & Evaluating	Quantitative and qualitative feedback about the progress and quality of implementation accompanied with regular personal and team debriefing about progress and experience		*Not assessed*	*I have access to data to help track changes in outcomes*	**[NO CHANGE from version used in Think Aloud interviews]** *I have access to data to help track changes in outcomes*

^a^The pCAT was iteratively updated based on input from interviewees as interviews were completed

### Think Aloud method

**Fig. 1:**
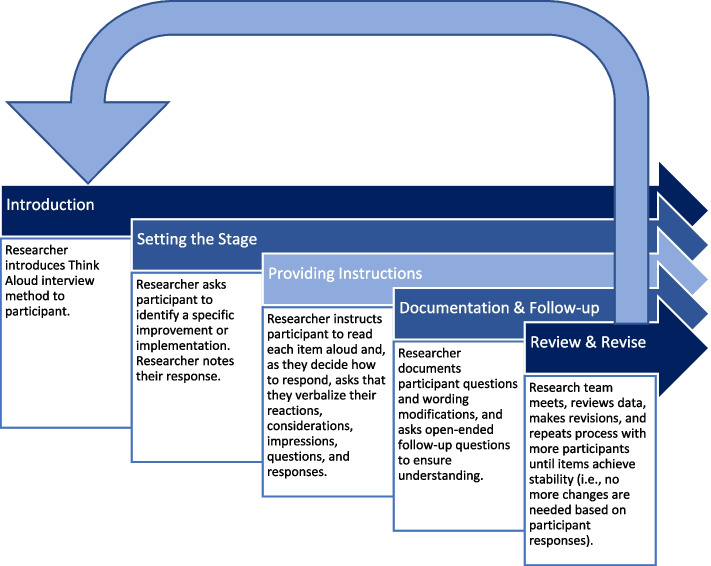
Think Aloud interview procedure

The updated version of the pCAT (see Table 1) was incorporated into the interview guide with the goal of engaging individuals using a Think Aloud method [24] that asks participants to verbalize their thoughts as they consider how to respond to questions in the assessment tool. Specifically, as participants responded, we asked them to verbalize their considerations, interpretations, and to ask questions or seek clarifications, if needed. We encouraged participants to verbally identify areas of disconnect, misinterpretation, and misunderstanding with the language and concepts being used. Interviewees were instructed to read each item out loud and say out loud, everything that came to mind. This included thoughts about the CFIR construct itself, the formatting of the tool, the language used to frame each construct, and their actual response as it related to their local quality improvement context. Interviewees were informed that the interviewer may periodically ask follow-up questions but capturing stream-of-consciousness interpretation of the tool was the primary goal. Iterative changes to the pCAT tool were made based on interviewee feedback (see Fig. 1).

### Participants

Participants included members of teams that participated in the LEAP quality improvement learning program after its initial pilot. Potential participants were invited to a telephone interview approximately 6 months after completing LEAP.

### Interviews

Interviews lasted for about an hour and were conducted from March 2018 through August 2019, audio recorded, and transcribed verbatim.

### Coding and analysis

Qualitative descriptions of barriers and facilitators in the transcripts were coded using CFIR constructs as preliminary codes. Additional codes were developed to capture more specificity when needed (e.g., adding consideration of Time as a subconstruct of Available Resources). As each interview was completed, language in the pCAT was iteratively updated as needed, based on input from each participant.

NVivo 12 Pro was used to facilitate coding [25]. Interviews were conducted by CHR. CHR and LJD examined early interview transcripts independently and participated in consensus discussions to establish initial coding and preliminary findings; all subsequent coding and iterative updates of the pCAT were done by (CHR) [26]. The Consolidated Criteria for Reporting Qualitative Studies checklist was used to guide the reporting of data collection and analysis activities [27].

### Human protections

This work was developed as a non-research activity (i.e., without Institutional Review Board approval under the authority of Veterans Health Administration (VHA) operations) and complies with the guidance about authorization of non-research manuscripts outlined in VHA Program Guide 1200.21: VHA Operations Activities That May Constitute Research [28]. All authors attest that the activities that resulted in the production of this manuscript were conducted as part of the non-research activities conducted under the authority of the VHA National Center for Health Promotion and Disease Prevention.

## Results

Thirty-eight invitations were sent to individuals on 34 teams that participated in LEAP after the initial pilot; 27 interviews were completed (71% response rate). Two interviews included two individuals from the same team at their request; the rest were one-on-one. The average length of the interviews was 47 min (range 27–63 min); all participants successfully completed their interview. Additional file 1 contains the final version of the abbreviated pragmatic context assessment tool (pCAT) based on results from interviews. The pCAT evolved as interviews progressed, based on experiences and input from the first nine people interviewed; the remaining 18 people did not express any challenges in responding to questions and their responses were in line with the intent of each question, indicating stability of the tool. The following sections highlight key themes that influenced changes made to the context assessment tool.

### Specificity of the change: question stem

The first task for participants was to describe the change or improvement being implemented. Initially, the guidance was, “*Please enter your problem area (area for improvement). This should reflect whatever topic you and your team are currently considering. It does not have to be final (e.g., The majority of patients fail to show up for scheduled orientation)*”. However, participants found this guidance too broad and speculative, and they struggled to provide assessments. It was easier for participants when they anchored their responses to a specific, recent, or on-going improvement or implementation effort as they considered each construct. Participants observed that each construct could be a facilitator with one improvement effort and a barrier with another, affirming that context *and* knowing what the change is, matters. For example, communication may be a facilitator when the implementation involves people from the same service line but becomes a barrier when the change requires communication and cooperation across service lines. Attempting to rate CFIR constructs was much more difficult and far less useful than critically assessing the specific context of a specific planned or on-going implementation.

Thus, we edited the “stem” to be more specific and concrete. The final guidance was developed as, “*We’ve found that it’s best to think concretely about a planned or on-going implementation (as opposed to the more general implementation environment). Include the specifics of the implementation/improvement project here.*” We allowed flexibility in interpretation of “changes” as “implementation” or “improvement” because both involve implementing a planned change.

### Identifying barriers versus facilitators

For each construct, participants were asked whether they agreed or disagreed with each statement. Agreeing meant the construct was a facilitator and disagreeing meant that the construct was a barrier. Participants could also be “neutral.” However, participants had difficulty indicating a level of agreement and instead wanted to answer with yes/no. To address this, we added explanatory text for Agree (this means the item is a potential facilitator) and Disagree (this means the item is a potential barrier). This change helped participants respond more accurately.

### Response options

After introducing explanations for assessing constructs as barrier versus facilitator (or neutral), participants were asked to assess the potential impact on implementation. Choices included three levels of impact (low, moderate, and high). Participants had difficulty differentiating between three levels and understanding how to assess impact (or influence). They were more comfortable assessing the effect (or consequence). Thus, we simplified responses to include “Weak/no effect” and “Strong effect” options.

### CFIR construct assessments

Six of ten CFIR constructs in the final version of the pCAT were unchanged from the version initially used in the think-aloud interviews (Patient Needs & Resources, Networks & Communications, Compatibility, Goals & Feedback, and Reflecting & Evaluating). The remaining four CFIR constructs shifted from future focus (e.g., “we will have…”) to current state (e.g., “we have…”). Additional changes are described below.

#### Relative advantage and tension for change

References to “key people” in these constructs were too vague for respondents. We revised language to refer to “people here” so respondents could tailor respond based on their knowledge of people most relevant for assessing relative advantage; this appeared to resolve difficulties in subsequent interviews.

#### Leadership engagement

The pCAT initially had a single question about “leaders here.” Participants had difficulty responding to this question without first considering the levels and types of leaders they work with, who may or may not have been involved in the improvement and then determining what they knew about their respective degree of engagement. Based on this feedback, we split CFIR’s “Leadership Engagement” construct to include two levels of leadership: (1) “leaders I work with most closely” and (2) “higher level leaders.” This change enabled respondents to respond more accurately.

#### Available resources

The pCAT Version 1.0 included a single question about “Available Resources.” Based on LEAP coach experiences with LEAP teams prior to our Think Aloud interviews, we separated this single question into three separate questions in pCAT Version 2.0. With this change, respondents had no difficulty answering separate questions about time and space. For “other needed resources,” respondents revealed a range of resources that might be needed including incentives for program participants and having a discretionary budget. Version 2.0 also incorporated current-state language instead of future-focused language as described above.

### Other suggested improvements

Participants were asked about any additional barriers or facilitators. One participant suggested asking about longer-term sustainment instead of focusing on short-term change. Another participant suggested adding open-text space to allow respondents to explain and justify their responses and to reflect on variation or disagreement among team members.

## Discussion

Our Think Aloud approach engaged frontline clinicians in the process of developing an abbreviated practical context assessment tool using plain language. The pCAT comprises 14 questions that assess ten CFIR constructs that range across four of the five framework domains: Innovation Characteristics, Outer Setting, Inner Setting, and Process (a copy is provided in Additional file 1). These constructs are among the most frequently reported as key determinants of implementation outcomes using the CFIR [2, 29]. Some of these constructs are also important for Lean quality improvement principles such as Goals and Feedback (i.e., alignment with objectives), Reflecting and Evaluating (e.g., using data to track outcomes), and Networks and Communications (e.g., open lines of dialogue) [30].

Context assessments are rarely done by practitioners within their own setting [31]. One reason for this is that measurement instruments often require expertise and are burdensome to apply [18, 31]. In deference to expertise and knowledge of frontline clinicians within their own setting [32] and in acknowledgement of their limited time, practical context assessment tools are needed that provide brief ratings of context to generate reflection and problem-solving by frontline teams engaged in improvement and that may help increase response rates for researchers and implementers who rely on these assessments to design strategies for successful implementation [1, 33].

Stanick et al. developed objective criteria by which to assess pragmatism of a measurement instrument [18], dividing criteria into “stakeholder-facing” and “objective” criteria. We applied each of the five objective criteria, which each use a six-point rating scale (− 1 to + 4; see Table 2). Based on these objective criteria, the pCAT is relatively pragmatic with scores of + 3 or + 4 for all criterion.

**Table 2:** Objective pragmatic rating criteria

Criteria	Rating^a^
Acceptability category	
Cost	4—Excellent: The measure is free and in the public domain
Easy category	
Uses accessible language	3—Good: The readability of the measure is between an 8th and 12th grade level
Assessor burden (training)	4—Excellent: The measure requires no training and/or has free automated administration
Assessor burden (interpretation)	3—Good: The measure includes a range of scores with value labels and cut-off scores, but scoring requires manual calculation and/or additional inspection of response patterns or subscales, and no instructions for handling missing data are provided
Length	3—Good: The measure has greater than 10 items but fewer than 50

These items only include PAPERS^18^ items related to objective characteristics of measurement instruments. The PAPERS instrument also includes “stakeholder-facing” criteria based on user ratings (e.g., usefulness) that were not assessed

^a^Rating scale is − 1 to + 4

The pCAT is available online [30]. It requires no specialized training to administer and can be completed electronically or on paper. The pCAT has limitations. First, this tool is an abbreviated assessment and is not designed to comprehensively assess all CFIR constructs. Though construct coverage is limited, those included align with the updated version of the CFIR [34]. The pCAT does not provide guidance about what respondents should do with the information elicited. Within the LEAP program [23], coaches worked with teams and highlighted the value of identifying barriers and facilitators when implementing changes so that barriers can be avoided or minimized and facilitators can be leveraged for success. Waltz et al. list recommended strategies that may best address each CFIR construct that manifests as a barrier [1]. Table 3 lists implementation strategies with the highest rate of endorsement for each of the ten constructs in pCAT that could be considered. Another key limitation of the pCAT is that each CFIR construct is assessed with a single question and does not follow a psychometric paradigm of development. The pCAT is offered as a brief practical tool for use by frontline teams and or coaches or facilitators to encourage collective understanding of local barriers and facilitators and to generate discussion about potential strategies based on this information. Content and structure of the final version is based on experiences of 27 individuals who were engaged in a quality improvement learning program. All respondents were frontline clinicians who were members in quality improvement teams embedded in a VHA medical center-based weight management program.

**Table 3:**
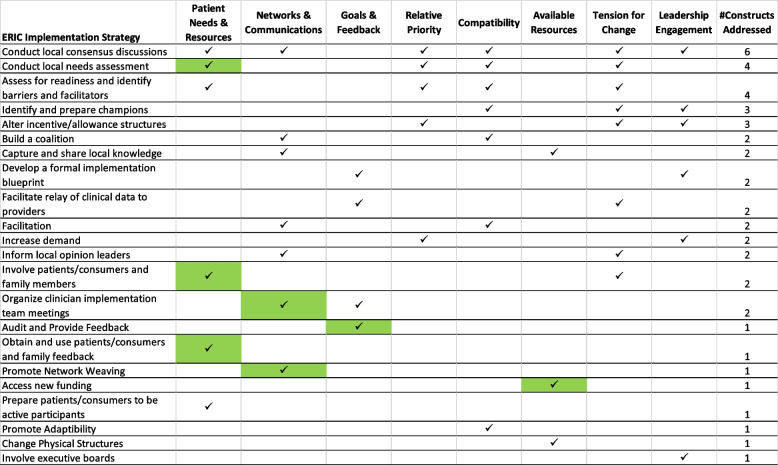
List of implementation strategies recommended to address pCAT constructs

ERIC implementation strategy names are from Powell et al. 2015 [35]

Shaded cells indicate strategies with the strongest endorsement by self-described implementation experts; unshaded checkmarks indicate strategies with at least 20% of implementation experts endorsing that strategy

## Conclusion

The pragmatic context assessment tool (pCAT) is designed as an abbreviated pragmatic approach to assess barriers and facilitators in clinical settings. It is short (14 items), available online (www.cfirguide.org), and is designed to draw on the expertise and knowledge of people who work at the frontline and are most familiar with their own clinical context.

## Supplementary Information

**Additional file 1.**

**Additional file 2.**

## Data Availability

The datasets generated and/or analyzed during the current study are not publicly available because qualitative and quantitative data are highly processed to support this study and to protect the identity of the individuals and locations who participated in the study. These data are, however, available from the corresponding author on reasonable request.

## Abbreviations

CFIR: Consolidated Framework for Implementation Research
LEAP Program: Learn. Engage. Act. Process
pCAT: Pragmatic context assessment tool
VHA: Veterans Health Administration
