# Supplementary material for: A pragmatic context assessment tool (pCAT): using a Think Aloud method to develop an assessment of contextual barriers to change
Source: Implement Sci Commun. 2023 Jan 11;4:3. doi: 10.1186/s43058-022-00380-5 (PMC9835384; doi:10.1186/s43058-022-00380-5)
Supplement: Supplementary file 1 — Additional file 1. [file 43058_2022_380_MOESM1_ESM.docx]

**Supporting Qualitative Quotes**

| **pCAT Element** | **Quote** |
| --- | --- |
| Specificity of the Change: Question Stem | P: “…So, I don't know that I’m going to help you with my answer, but I’m kind of neutral on this because it’s dependent on what that change is, implementing it versus an alternative. So, I’m kind of neutral on that question”. (ID 74001) |
| Identifying Barriers versus Facilitators | *P: “Okay, let’s start now. “People here regularly seek to understand the needs of patients and make changes to better meet the needs of patients,” I will say, yes for the most part, yes”. (ID 52801)* |
| Assessing Impact on Implementation | *I: And then what is the likely impact of this factor of space on implementing your 12-week*  *program?*  *P: So, when you say moderate impact, is that, are you saying it’s like a negative impact or a positive impact?*  *I: Well so this is a good question, what one of those comes to mind first when you read the options there?*  *P: So, I usually think of low impact meaning it has like minimal effect versus high impact having high or positive effect. So, when you say sufficient space to accommodate the change, yeah, I would kind of rate it as moderate impact.* (ID 32401) |
| Relative Advantage and Tension for Change | *I: …“Key people will see the advantage of implementing*  *the change versus an alternative.”*  *P: Okay and key people, is that kind of a loose definition?*  *I: I’d like you to define key people for me.*  *P: So, I think the key people, I mean I kind of think first*  *and foremost the key people would be*  *the participants [...] The other key people may be our own*  *team and referring providers […]*  (ID 32401) |
| Leadership Engagement | *P: “Leaders I work with most closely are committed, involved, and accountable for the planned improvement, okay so by leaders, people that I report to are leaders? Like up the chain? Like my Service Chief? Chief of other services? I guess the term leaders is kind of open there. So, I would say, it's hard to say, as a whole, all leaders. I mean of course the people that I work, you know the leaders that I work with, I feel they are committed, and they are held accountable for the work that they do”. (ID 30801)* |
| Other Suggested Improvements | *P: “…I don't know if it would go in this tool, but I guess like*  *sustainability where it’s easy to make a change for this X*  *amount of time, but then to continue the change when*  *you're not gathering data, I don't know, I guess I'm kind*  *of thinking about sustainability of the change. I guess this*  *could also go under policy, like if there’s not a policy on it,*  *then you're not going to be held to keep doing it, but I*  *guess I don't really necessarily think the policy question*  *quite came to that…” (ID 85001)* |
|  | P: *“…Do you remember earlier I said sometimes you feel like you’re boxed in with questions? [I: Yes] I feel like I’m almost boxed in. Like there would be, on some of these questions I would have more to say, and I don’t have the ability to say it anywhere, so it’s kind of like, I don't know, if I were doing this questionnaire with a group of folks, I would get the information, to some extent it would be helpful, but I wouldn’t have answers to all my questions. Does that make sense?” (ID 74001)* |
|  | *P: “…if a situation is intolerable, what do people see as intolerable? If they agree that it’s important, you know the facilitator has a strong effect, well okay, so now what? You know? Because what I interpret as intolerable may not be somebody else’s they may not mean what I’m thinking, …so I need more details”. (ID 74001)* |
