# Supplementary material for: A pragmatic context assessment tool (pCAT): using a Think Aloud method to develop an assessment of contextual barriers to change
Source: Implement Sci Commun. 2023 Jan 11;4:3. doi: 10.1186/s43058-022-00380-5 (PMC9835384; doi:10.1186/s43058-022-00380-5)
Supplement: Supplementary file 2 — Additional file 2. [file 43058_2022_380_MOESM2_ESM.docx]

**Barrier Buster Tool**

We have developed this abbreviated 14-item measure that assesses constructs that are most commonly associated with outcomes within our own implementation work. Our goal with this tool was to create a way to assess a selected number of CFIR constructs using everyday language. We used a “think aloud” process with users to refine wording and items.

This tool is designed to help teams identify barriers and facilitators at various points throughout the implementation process. The Barrier Buster Tool focuses on implementation constructs commonly present in successful implementations. Anticipating potential barriers allows teams to strategize ways to overcome, minimize, or bypass them. Identifying facilitators allows teams to strategize ways to maximize their effects. You may consider using this tool during the initial planning phase and again mid-implementation. We’ve found that it’s best to think concretely about a planned or on-going implementation (as opposed to the more general implementation environment).

| **Improvement to consider (include the specifics of the implementation/improvement project here):** |
| --- |

|  | Indicate your agreement with this statement:  **1 – DISAGREE**: This means the item is a potential **barrier**  **2 – Neutral**  **3 – AGREE:** This means the item is a potential **facilitator** | This **barrier** will have…  This **facilitator** will have… | What is the likely effect of this barrier/facilitator on your ability to implement the improvement?  0 – Weak/no effect  1 – Strong effect  0 – Weak/no effect  1 – Strong effect |
| --- | --- | --- | --- |
| 1. People here regularly seek to understand the needs of patients and make changes to better meet those needs. (**Patient Needs & Resources)** |  |  |  |
| 1. I have open lines of communication with everyone needed to make the change. (**Networks & Communications)** |  |  |  |
| 1. I have access to data to help track changes in outcomes. **(Reflecting & Evaluating)** |  |  |  |
| 1. The change is aligned with leadership goals. **(Goals & Feedback)** |  |  |  |
| 1. The change is aligned with clinician values. **(Compatibility)** |  |  |  |
| 1. The change is compatible with existing clinical processes. **(Compatibility)** |  |  |  |
| 1. The structures and policies in place here enable us to make the change. **(Structural Characteristics)** |  |  |  |
| 1. We have sufficient space to accommodate the change. **(Available Resources)** |  |  |  |
| 1. We have sufficient time dedicated to make the change. **(Available Resources)** |  |  |  |
| 1. We have other needed resources to make the change (staff, money, supplies, etc.). **(Available Resources)** |  |  |  |
| 1. People here see the current situation as intolerable and that the change is needed. **(Tension for Change)** |  |  |  |
| 1. People here see the advantage of implementing this change versus an alternative change. **(Relative Advantage)** |  |  |  |
| 1. Higher level leaders are committed, involved, and accountable for the planned improvement. **(Leadership Engagement)** |  |  |  |
| 1. Leaders I work with most closely are committed, involved, and accountable for the planned improvement. **(Leadership Engagement)** |  |  |  |

| Additional notes/thoughts/details: |
| --- |
